# Bisphenol S Induces Lipid Metabolism Disorders in HepG2 and SK-Hep-1 Cells via Oxidative Stress

**DOI:** 10.3390/toxics13010044

**Published:** 2025-01-08

**Authors:** Kai-Xing Lin, Zi-Yao Wu, Mei-Lin Qin, Huai-Cai Zeng

**Affiliations:** 1 Guangxi Key Laboratory of Environmental Exposomics and Entire Lifecycle Health, School of Public Health, Guilin Medical University, Guilin 541199, China; linkaixing922@163.com (K.-X.L.); ziyaowu@163.com (Z.-Y.W.); qmeilin2022@163.com (M.-L.Q.); 2 Guangxi Health Commission Key Laboratory of Entire Lifecycle Health and Care, School of Public Health, Guilin Medical University, Guilin 541199, China

**Keywords:** oxidative stress, lipid metabolism, bisphenol S, metabolic disorders

## Abstract

Bisphenol S (BPS) is a typical endocrine disruptor associated with obesity. To observe BPS effects on lipid metabolism in HepG2 and SK-Hep-1 human HCC cells, a CCK-8 assay was used to assess cell proliferation in response to BPS, and the optimal concentration of BPS was selected. Biochemical indices such as triglyceride (TG) and total cholesterol (T-CHO), and oxidative stress indices such as malondialdehyde (MDA) and catalase (CAT) were measured. ROS and MDA levels were significantly increased after BPS treatment for 24 h and 48 h (*p* < 0.05), indicating an oxidative stress response. Alanine aminotransferase (ALT), T-CHO, and low-density lipoprotein cholesterol (LDL-C) levels also increased significantly after 24 or 48 h BPS treatments (*p* < 0.05). RT-PCR and Western blot analyses detected mRNA or protein expression levels of peroxisome proliferator-activated receptor α (PPARα) and sterol regulatory element-binding protein 1c (SREBP1C). The results indicated that BPS could inhibit the mRNA expression of PPARα and carnitine palmitoyl transferase 1B (CPT1B), reduce lipid metabolism, promote mRNA or protein expression of SREBP1C and fatty acid synthase (FASN), and increase lipid synthesis. Increased lipid droplets were observed using morphological Oil Red O staining. Our study demonstrates that BPS may cause lipid accumulation by increasing oxidative stress and perturbing cellular lipid metabolism.

## 1. Introduction

Bisphenol S (BPS) serves as a substitute for BPA and is incorporated in a variety of industrial and consumer products [1]. It exerts endocrine-disrupting effects and can seriously affect the endocrine system in various ways [2], leading to lipid metabolism disorders and serious damage to the human body [3]. The median value of BPS glucosinolates detected in the urine of the general population was 0.38 ng/mL [4], and the median urinary BPS concentration in the occupational population of cashiers exposed to thermal paper was 2.53 μg/L [5], much higher than that of the general population. During the seawater monitoring in the Pearl River Estuary in China, BPS was observed at a detection rate of 100%, and this was similar to that of BPA and BPF [6]. However, during the detection of biodegradation of bisphenol compounds in seawater, BPS exhibited higher stability and more difficult biodegradation than BPA and BPF [7]. Due to increasing levels of BPS exposure in the environment, its safety has attracted increasing attention.

Obesity has become increasingly prevalent. BPS is a typical endocrine disruptor closely linked to the occurrence of obesity [8]. Studies have demonstrated that BPS promotes weight gain in adults [9], and it is significantly associated with obesity [10]. Meng et al. [11,12] reported that BPS exposure may disrupt lipid and glucose metabolism in perinatal male and female mice, enhancing fatty acid and triglyceride (TG) production and elevating obesity risk. Another study indicated that BPS caused hepatic steatosis and lipid accumulation in mice as well as oxidative stress [13]. Serum BPS levels in humans are connected to oxidative stress and endocrine disorders [14]. Prior research has indicated that BPS increases reactive oxygen species levels in adipocyte progenitors and promotes fat synthesis [15]. Chen et al. [16] observed that improving oxidative stress can alleviate type 2 diabetes combined with non-alcoholic fatty liver disease.

In regulating the mechanism of liver lipid metabolism disorders, the PPAR signaling pathway plays an important role. For example, peroxisome proliferator-activated receptor α (PPARα) is a key regulator of liver beta-oxidation, which is involved in the main process of fatty acid metabolism in the liver, and it regulates downstream target genes, such as carnitine palmitoyl transferase 1 (CPT1) [17], to control mitochondrial fatty acid beta-oxidation. Overexpression of the novel biomarker for non-alcoholic fatty liver, MIR20B, significantly reduces the oxidation of free fatty acids by targeting PPARα, resulting in a significant upregulation of TG and cholesterol content in HepG2 cells treated with oleic acid [18]. Qiu et al. [19] demonstrated that BPS exposure induces chronic inflammatory stress in the liver. When carp livers were exposed to 100 mg/L of both BPS and BPA, the oxidative stress levels induced by BPS were significantly higher compared to those induced by BPA. This also illustrates that BPS is not necessarily a safe alternative to BPA and that its potential harm to organisms or the environment should be further studied. Current evidence suggests that BPS may affect the gene expression of liver lipid synthesis, transport, and metabolism through oxidative stress, leading to lipid accumulation and alleviating oxidative stress in HepG2 cells by affecting the sterol regulatory element-binding protein 1c (SREBP1C)/FASN pathway through PPARα [20]. Therefore, this study mainly examined proteins or genes related to liver lipid synthesis and metabolism, such as SREBP1C, fatty acid synthase (FASN), PPARα, carnitine palmitoyl transferase 1B(CPT1B), etc.

The impact of BPS on lipid metabolism in HepG2 and SK-Hep-1 cells remains unclear. Current research indicates that BPS causes oxidative stress, which alters the expression of genes in the liver linked to lipid production, transport, and metabolism and results in lipid accumulation. In vitro studies on human red blood cells revealed no discernible alteration in the levels of catalase (CAT) after treatment with 100 μg/mL BPS for 24 h [21]. Considering that different individuals exhibit different sensitivities to chemicals and that the HepG2 cell line’s metabolome is unstable, a major transition may occur over time [22]. To this end, this study utilized the HepG2 and SK-Hep-1 human hepatocellular carcinoma cells to assess the responses to BPS treatment for 24 h and 48 h, respectively. A BPS concentration of 250 μmol/L was selected according to the results of CCK-8 experiments. Based on the detection of malondialdehyde (MDA), TG, total cholesterol (T-CHO), and other indicators, the staining results of Oil Red O assays, and reactive oxygen species assessments, the aim is to examine how lipid metabolism is affected by varying BPS treatment durations in the experimental cell lines, thus providing a reference for the study of the mechanisms of preventing environmental pollutants from causing obesity by inducing lipid accumulation.

## 2. Experimental Method

### 2.1. Cell Culture and Exposure

For cell culture, human hepatocellular carcinoma cells (HepG2 and SK-Hep-1) were rapidly thawed and inoculated into culture dishes containing 7 mL of complete medium and were incubated at 37 °C under 5% CO_2_. Cell exposure refers to the transfer of two separate dishes of 80% grown cells to 10 mL centrifuge tubes, the addition of 6 mL of complete medium, the inoculation of cells into 6-well plates with a 0.5 mL density of cell volume per well, and the replacement of the complete medium with new complete medium when growth reaches 50%. After 24 h of BPS exposure, each well was filled with 3 mL of complete medium, and after 48 h of BPS exposure, each well was filled with 4 mL of complete medium. According to whether the cells were exposed to BPS or not, the two types of cells were divided into control and BPS-stained groups, respectively. An amount of 3 or 4 μL of DMSO was incorporated into the control group, and 3 or 4 μL of the 250 mmol/L BPS (Sigma, Shanghai, China) master batch was added to the BPS group, which ensured that the BPS was exposed at a final concentration of 250 μmol/L for 24 or 48 h. The final BPS concentration for cell exposure was 250 μmol/L.

### 2.2. CCK-8 Method Cell Viability Assays

HepG2 and SK-Hep-1 cells under optimal growth conditions were prepared into single cell suspensions, and 10 μL was extracted for cell counting to adjust the number of cells. After 24 or 48 h BPS exposure, HepG2 cells were inoculated at a density of 3000 or 2000 cells per well, and SK-Hep-1 cells were inoculated at a density of 4000 or 3000 cells per well. After 50% of the cells adhered to the wall, the BPS stock solution was diluted into 0, 50, 150, 200, 250, 300, and 350 μmol/L solutions with complete medium and then added to each well at a total volume of 100 μL. The treated cells were cultured in a CO_2_ incubator (Thermo Fisher Scientific, Waltham, MA, USA) for 24 or 48 h, and the 96-well plates were then removed to discard the original medium. Each well was then filled with complete medium with 10 μL of CCK-8 solution at a 10:1 ratio. Following a one-hour incubation period at 37 °C, absorbance values in each well were measured at 450 nm.

### 2.3. ROS Reactive Oxygen Detection

After cell treatments, the DCFH-DA probe was diluted to 10 μmol/L with preheated serum-free medium according to the instructions of the reactive oxygen species detection kit (Beijing Solarbio Science & Technology Co., Ltd., Beijing, China, CA1410). The original medium was removed, and the cells underwent two gentle PBS washes, were injected with DCFH-DA probe dilution solution in the dark and were incubated for 20 min at 37 °C in a cell incubator. Serum-free cell culture media was then used to wash the cells three times. After removing residual DCFH-DA, the cells were observed and captured on camera using an inverted microscope.

### 2.4. Oil Red O Staining

After the cells were exposed to BPS and cultured, they were washed twice with PBS buffer, fixed by adding Oil Red O (Beijing Solepol Science and Technology Co., Ltd., Beijing, China, G1262) fixative for 20 min, and the fixative was discarded and washed twice with distilled water. The process of Oil Red O staining was carried out sequentially according to the following steps: 60% isopropanol wash for 20 s, Oil Red O dip staining for 10 min, 60% isopropanol wash for 20 s, distilled water wash 3 times, hematoxylin staining for 1 min, distilled water wash 3 times, and dip staining with Oil Red O buffer for 1 min. Finally, after discarding the Oil Red O buffer, distilled water was added to cover the cells, and the lipid droplet production of the cells in each group was observed under an inverted microscope.

### 2.5. Measurement of Biochemical Indicators and Oxidative Stress Indicators

Because HepG2 cells and SK-Hep-1 cells are different in the 6-well plate, different levels of lysate are required. After cell exposure culture, 400 μL of cell lysate was added to a 6-well plate containing HepG2 cells, and 300 μL of cell lysate was added to a 6-well plate containing SK-Hep-1 cells for protein extraction. Intracellular TG (A110-1-1), T-CHO (A111-1-1), low-density lipoprotein cholesterol (LDL-C, A113-1-1), alanine aminotransferase (ALT, C009-2-1), malondialdehyde (MDA, BC0025), catalase (CAT, C009-1-1), and T-CHO (A111-1-1) were measured according to the kit’s instructions. Following the detection of the indicators, a full wavelength scanning multifunction microplate reader (Thermo Scientific Varioskan LUX, Waltham, MA, USA) was used to quantify each kit. Every kit was acquired from the Nanjing Institute of Bioengineering, China.

### 2.6. Real-Time Quantitative PCR (qPCR) Analysis

TRIzol reagent (Ambion, Austin, TX, USA) was used to extract total RNA and determine its concentration, and reverse transcription was used to synthesize cDNA using a reverse transcription kit (Monad, Wuhan, China). The mRNA expression levels of PPARα, CD36, SREBP1C, and FASN were detected using a QuantStudio 6 Flex according to the instructions for the reagent. The primer sequences are presented in Table 1.

### 2.7. Western Blotting

After extracting the cell protein, the BCA kit (Beyotime, Shanghai, China) was used to determine the cell protein content and adjust samples to the same concentration, and the protein sampling buffer was added and then heated at 100 °C for 5 min to prepare the protein samples. A total of 10 μL of the protein samples was aspirated and added to the concentrated gel wells, and after electrophoresis at 80 V to move the proteins to the separation gel, the voltage was increased to 120 V for 1 h. After transferring the protein to polyvinylidene fluoride, it was shaken at room temperature for 1 h, followed by the addition of a rapid containment solution, thus allowing the protein primary antibodies of sterol regulatory element binding protein (SREBP1C, 1:2500, Proteintech Group, Inc., Wuhan, China), fatty acid synthetase (FASN, 1:1000, servicebio, Wuhan, China), and GAPDH (1:2000, servicebio, Wuhan, China) to be incubated at 4 °C overnight. The following day, goat anti-rabbit or goat anti-mouse secondary antibodies were added to the solution, incubated for 1 h at room temperature and finally developed using a gel-imager.

### 2.8. Statistical Analysis

IBM SPSS Statistics 21 software and ImageJ were used for statistical analysis. Images were drawn using PRISM 8.0 and Adobe Illustrator CS6. The data are expressed as mean ± standard error ($\bar{x}$ ± s), and the test level was set as α = 0.05.

## 3. Results

### 3.1. Selection of the Best BPS Concentration

**Figure 1:**
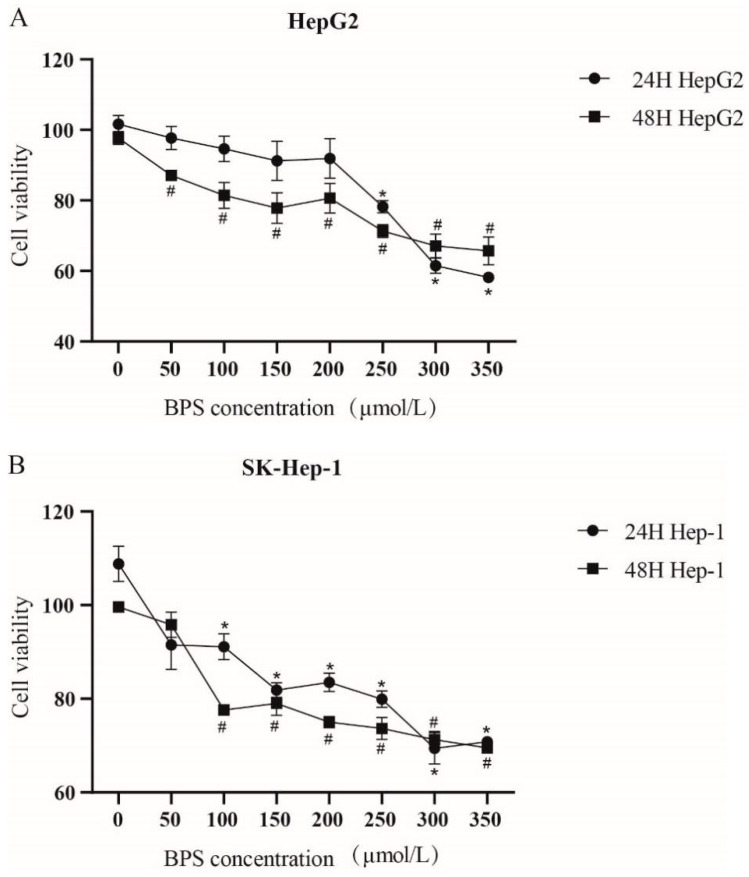
Effect of different concentrations of BPS on the viability of HepG2 and SK-Hep-1 cells. Note: (**A**,**B**) presents the alterations in cell viability following treatment with varying concentrations of BPS. “*” represent cell viability following a 24 h exposure to BPS relative to the 0 μmol/L group, *p* < 0.05. “#” represents cell viability following a 48 h exposure to BPS relative to the 0 μmol/L group, *p* < 0.05. n = 3, the same below.

Cell viability was measured by CCK-8 assay after 24 h and 48 h BPS treatment. With an increase in the exposure concentration, the survival rate of HepG2 and SK-Hep-1 cells diminished in a dose-dependent fashion (Figure 1A,B). At 250 μmol/L, the cell viability of HepG2 and SK-Hep-1 was between 70% and 80%. To simultaneously study the effect of BPS on HepG2 cells and SK-Hep-1 cells and to ensure that BPS exerts an effect on cells without excessive damage, we selected the 250 μmol/L BPS concentration for subsequent experiments.

### 3.2. Effect of BPS on ROS Levels in HepG2 Cells and SK-Hep-1 Cells

**Figure 2:**
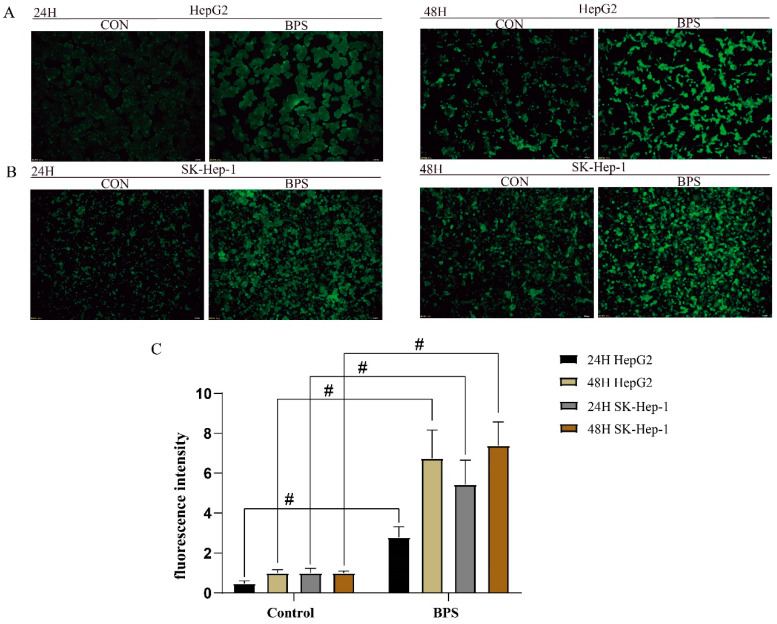
Effect of BPS treatment on ROS levels in HepG2 and SK-Hep-1 cells. Note: (**A**,**B**) shows the results of reactive oxygen species detection in HepG2 and SK-Hep-1 cells exposed to BPS for 24 h or 48 h conditions with a microscopic scale of 100 μm. (**C**) indicates reactive oxygen species fluorescence intensity quantification, and “#” represents the oxidative stress of cells after exposure compared to that of the control group, *p* < 0.05. n = 3.

After BPS treatment for 24 h, the BPS-treated HepG2 and SK-Hep-1 cells produced an obvious oxidative stress response, and the intensity of oxidative stress within the SK-Hep-1 cells was more obvious (Figure 2A,B). After 48 h of BPS treatment, oxidative stress in both cell types was markedly elevated compared to the control group. Notably, the degree of oxidative stress in HepG2 cells treated with BPS was stronger than that in the BPS group treated with BPS for 24 h. Therefore, an increase in BPS exposure time would increase the degree of oxidative stress in HepG2 cells.

### 3.3. BPS Induced Lipid Droplet Deposition in HepG2 and SK-Hep-1 Cells

**Figure 3:**
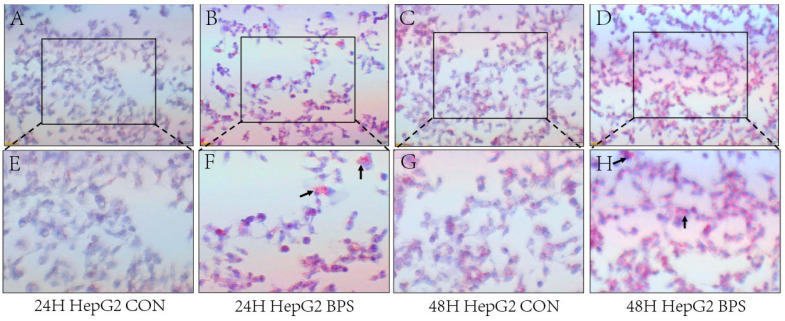
BPS-induced lipid droplet deposition in HepG2 cells. Note: (**A**–**D**) shows Oil Red O staining in HepG2 cells following BPS exposure for 24 h or 48 h with a microscopic scale of 50 μm. (**E**–**H**) presents the proportionally enlarged “□” window in (**A**–**D**), while “↑” in (**F**,**H**) refers to red fat droplets.

**Figure 4:**
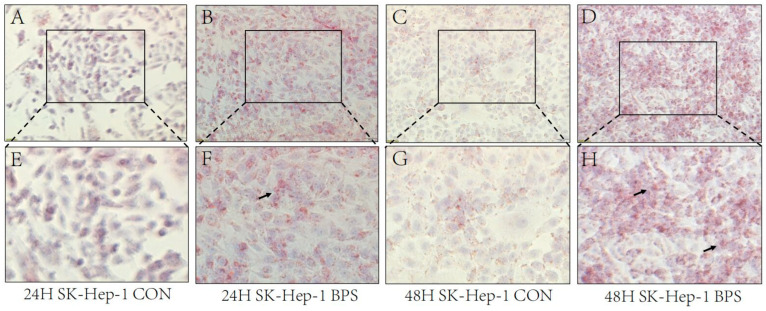
BPS-induced lipid droplet deposition in SK-Hep-1 cells. Note: (**A**–**D**) presents Oil Red O staining of SK-Hep-1 cells after exposure to BPS for 24 h or 48 h with a microscopic scale of 50 μm. (**E**–**H**) presents the proportionally enlarged “□” window in (**A**–**D**), while “↑” in (**F**,**H**) refers to the red fat droplets.

The effects of BPS on the intracellular lipid content in HepG2 and SK-Hep-1 cells were observed under a microscope. Both HepG2 and SK-Hep-1 cells produced red lipid droplets after 24 h and 48 h of exposure (Figure 3 and Figure 4A–H), and the production of lipid droplets in SK-Hep-1 cells exposed to BPS for 48 h was noticeably greater than that in the BPS group treated for 24 h.

### 3.4. Effect of BPS on MDA and CAT Levels in HepG2 and SK-Hep-1 Cells

**Figure 5:**
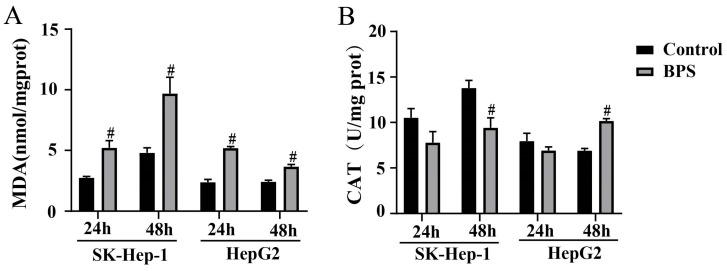
Effect of BPS on oxidative stress levels in HepG2 cells and SK-Hep-1 cells. Note: (**A**) shows the MDA levels within SK-Hep-1 and HepG2 cells following BPS treatments for 24 h and 48 h. (**B**) shows the CAT levels within SK-Hep-1 and HepG2 cells following BPS treatments for 24 h and 48 h; “#” represents BPS compared to the control group, *p* < 0.05. n = 3.

In contrast to the control group, a marked rise in MDA content was observed in both cells following 24 h and 48 h exposure (*p* < 0.05) (Figure 5A). The CAT content in SK-Hep-1 cells decreased after BPS treatment for 24 h; however, there was no statistically significant difference. After BPS treatment for 48 h, the CAT content significantly dropped (*p* < 0.05). The CAT content in HepG2 cells decreased after BPS treatment for 24 h, which was not statistically significant. But the CAT content increased significantly in HepG2 cells after BPS exposure for 48 h (*p* < 0.05) (Figure 5B).

### 3.5. Effects of BPS on HepG2 and SK-Hep-1 Cell Damage and Metabolic-Related Indicators

**Figure 6:**
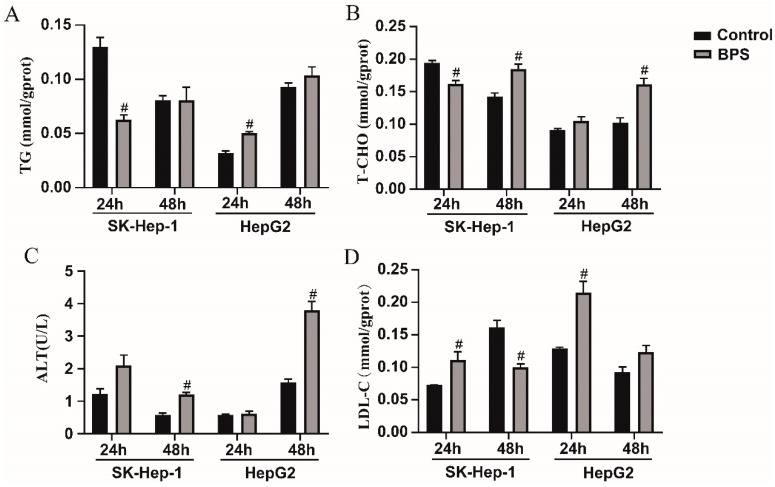
Effects of BPS on HepG2 and SK-Hep-1 cell damage and metabolic-related indicators. Note: (**A**–**D**) presents the results of TG, T-CHO, ALT, and LDL-C analyses after BPS exposure in SK-Hep-1 and HepG2 cells for 24 h and 48 h, respectively. “#” represents BPS compared to the control group, *p* < 0.05. n = 3.

After 24 h and 48 h of exposure, HepG2 cells’ levels of TG and T-CHO were noticeably greater than those in the control group (Figure 6A,B). However, after 24 h of exposure, the SK-Hep-1 cells exhibited significantly reduced TG and T-CHO levels compared to the control group (*p* < 0.05), and after 48 h of exposure, T-CHO levels were markedly higher compared to the control group (*p* < 0.05). At 24 h following exposure, no significant difference in ALT levels was observed between both cell types relative to the control group; however, at 48 h following exposure, the ALT levels were markedly elevated compared to the control group (Figure 6C). When exposed for 24 h, the LDL-C level in both cell lines was markedly greater than that in the control group, and after 48 h, the LDL-C level in SK-Hep-1 cells was lower than that in the control group. After 48 h of BPS exposure, there was no discernible variation between the control group and HepG2 cells (Figure 6D).

### 3.6. Effects of BPS on PPARα, CPT1B, CD36, SREBP1C, and FASN mRNA Levels in HepG2 and SK-Hep-1 Cells

**Figure 7:**
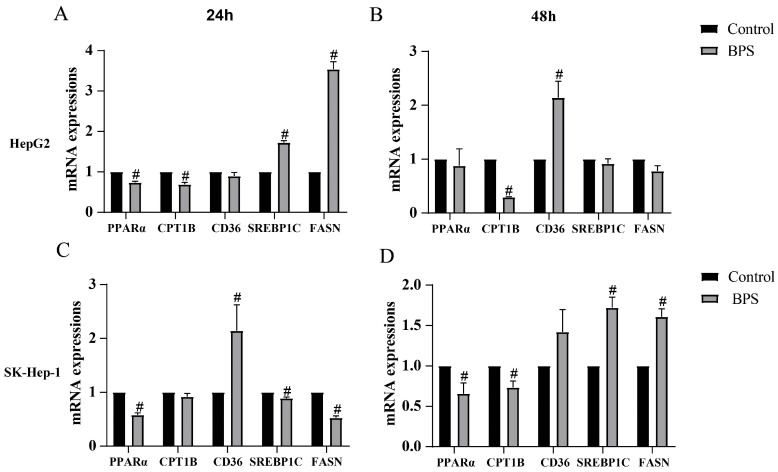
Effect of BPS on the expression levels of related mRNAs in HepG2 and SK-Hep-1 cells. Note: (**A**–**D**) indicates the mRNA expression results of PPARα, CPT1B, CD36, SREBP1C, and FAFSN in HepG2 and SK-Hep-1 cells following 24 h and 48 h of exposure, respectively. “#” represents BPS compared to the control group, *p* < 0.05. n = 3.

Following 24 h of BPS exposure, in contrast to the control, the mRNA expression levels of PPARα and CPT1B in HepG2 cells were significantly downregulated (*p* < 0.05), while the mRNA expression levels of SREBP1C and FASN were significantly upregulated (*p* < 0.05) (Figure 7A). SK-Hep-1 cells exhibited a marked decrease in the mRNA expression of PPARα, SREBP1C, and FASN, and there was a significant upregulation of CD36 mRNA expression (*p* < 0.05) (Figure 7C). After 48 h of BPS exposure, CPT1B mRNA expression in HepG2 cells was markedly reduced (*p* < 0.05) (Figure 7B), while the mRNA expressions of CD36 were significantly upregulated (*p* < 0.05) (Figure 7B). The mRNA levels of other genes did not change markedly. The mRNA expressions of PPARα and CPT1B in SK-Hep-1 cells were markedly downregulated, while the mRNA expressions of SREBP1C and FASN were significantly upregulated (*p* < 0.05) (Figure 7D).

### 3.7. Effects of BPS on the Expression Levels of the Lipid Synthesis Proteins SREBP1C and FASN in HepG2 Cells and SK-Hep-1 Cells

**Figure 8:**
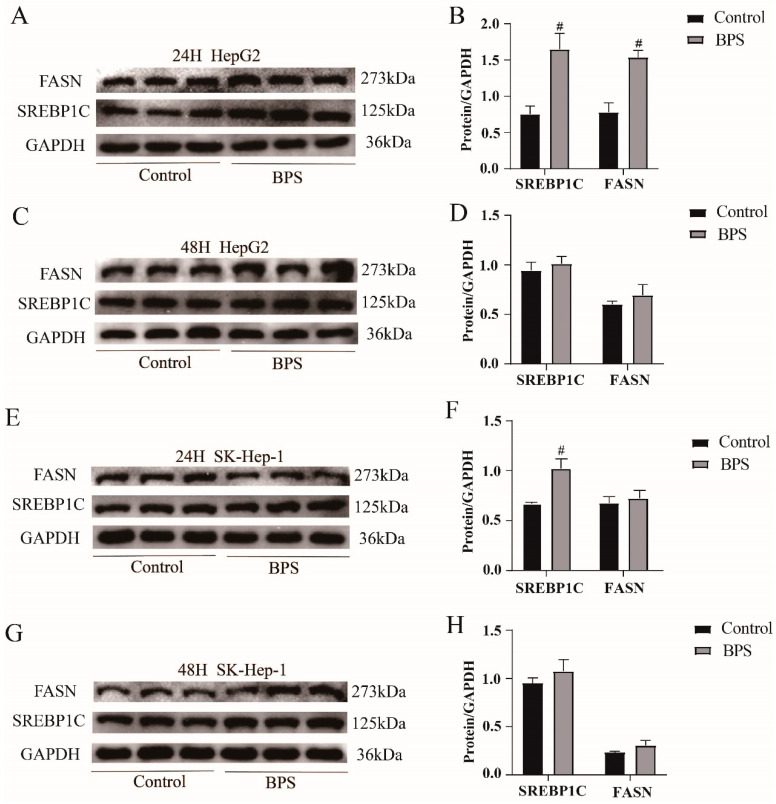
Effects of BPS on the expression levels of the lipid synthesis proteins SREBP1C and FASN in HepG2 and SK-Hep-1 cells. Note: (**A**–**H**) indicates the relative protein expression of SREBP1C and FASN in SK-Hep-1 and HepG2 cells after 24 h and 48 h of exposure, respectively. “#” represents BPS compared to the control group, *p* < 0.05. n = 3.

The protein expression levels of FASN and SREBP1C, which promote lipid synthesis, were found using Western blotting. After BPS treatment for 24 h, the protein expression levels of SREBP1C and FASN in the BPS group of HepG2 cells were markedly increased (*p* < 0.05) (Figure 8A,B). The expression of SREBP1C protein was markedly elevated (*p* < 0.05) in SK-Hep-1 cells (Figure 8E,F). There were no discernible alterations in HepG2 or SK-Hep-1 cells following 48 h of treatment (Figure 8C,D,G,H).

## 4. Discussion

Although BPS exerts endocrine-disrupting effects, the mechanisms underlying the metabolic disturbances triggered by BPS in hepatocytes remain unclear. Prolonged exposure to bisphenol compounds can increase cellular damage. When HepG2 cells were exposed to 0.625–10 μm BPS for 48 h, no or only slight cytotoxicity was produced, while after prolonged exposure, cytotoxicity was produced in a concentration-dependent manner [23]. The CCK-8 assay indicated that the viability of HepG2 and SK-Hep-1 cells gradually decreased after treatment with different concentrations (0, 50, 100, 150, 200, 250, 300, 350 μmol/L) of BPS for 24 or 48 h, indicating that BPS exerts a dose-dependent effect on cell damage. And increasing the concentration of the virus causes more obvious damage to cells. Studies have reported that oxidative stress leads to metabolic disorders and thus obesity [24] and that obesity can be alleviated by reducing and inhibiting liver oxidative stress and improving liver damage [25,26]. Obesity is often positively correlated with abnormal liver function. Zhang et al. [27] proposed that intracellular oxidative function is impaired in fatty liver lesions, and this can produce a large amount of ROS, leading to hepatocyte damage and the release of a large amount of ALT. Concurrently, AST levels increase with an increase in obesity [28], and antioxidant capacity also increases with an increase in TG and T-CHO levels [29]. Oxidative stress is both a precipitating factor and an endpoint of metabolic disorders [30,31]. Oral administration of BPS to mice at 5000 μg/kg/day or 50 mg/kg/day for 6–8 weeks resulted in liver damage, elevated ALT levels, and morphological changes to the liver [32,33]. In the present study, both the ROS content and MDA levels in HepG2 and SK-Hep-1 cells exposed to BPS for 24 h and 48 h were significantly increased, and the degree of oxidative stress in HepG2 cells was stronger after 48 h than 24 h of exposure to BPS, indicating that the cells not only developed oxidative stress, but also that the degree of oxidative stress might be enhanced by an increase in exposure time. The levels of ALT, T-CHO, and LDL-C in HepG2 and SK-Hep-1 cells treated with BPS for 24 h or 48 h significantly increased, including an increase in lipid droplets as assessed by Oil Red O staining, indicating that the cells were damaged to varying degrees after treatment with BPS for 24 h or 48 h. However, prolonged exposure to BPS increased lipid accumulation in HepG2 and SK-Hep-1 cells. In an analysis of a population-based study, elevated LDL-TG increased the risk of atherosclerosis, and it was therefore concluded that elevated LDL-TG could be used as a marker for atherosclerosis risk assessment [34]. Increased levels of ROS lead to oxidative stress in the vascular wall and support the oxidation of LDL, causing increased levels of ox-LDL, and when mitochondria are damaged by ROS accumulation, HDL and cholesterol metabolism will be defective [35]. This may be one of the reasons for increased levels of TG and T-CHO. Unexpectedly, after 24 h of BPS treatment, the TG content of SK-Hep-1 cells dropped, in contrast to HepG2 cells, and after 48 h, no discernible change was seen. According to previous studies, BPS increases leptin production in adipocytes [36]. However, the TG content of SK-Hep-1 cells decreased after BPS treatment, and whether the pathway promoting fat metabolism was activated remains to be further explored. Notably, the CAT content in SK-Hep-1 cells decreased after 24 h and 48 h of BPS exposure, but the CAT content in HepG2 cells increased after 48 h of BPS exposure, indicating that BPS caused cell damage and oxidative stress in both SK-Hep-1 and HepG2 cells. However, HepG2 cells demonstrated that antioxidant capacity increased with increasing TG and T-CHO levels, which is in line with the findings of earlier research [29]. In the study of Mohan et al. [37], antioxidant defense mechanisms were activated in freshwater fish for resistance to BPS-induced oxidative stress after exposure to BPS, and anti-oxidative stress indices such as superoxide dismutase (SOD) and catalase were significantly elevated. In one study [38], bovine granulosa cells showed a significant increase in SOD1 levels after 12 h exposure to 50 μg/mL BPS, and an increase in SOD2 levels at 48 h exposure, even though there was no significant change in CAT levels. This reflects the fact that cells are also more resistant to oxidative stress in the presence of oxidative stress. SOD1 and SOD2, which also have anti-oxidative stress effects, were overexpressed at different times of staining, which may be a compensatory response of cells against oxidative stress. In the present study, CAT levels in HepG2 cells were increased at 48 h of staining, perhaps also as a compensatory effect.

Lipid accumulation is a result of increased lipid synthesis and decreased lipid metabolism [39]. Our group has previously demonstrated that BPS induces heightened hepatic lipid synthesis and reduced hepatic lipid metabolism in C57 mice [33]. To verify whether this phenomenon is also present in liver cells, we examined the levels of lipid metabolism-related mRNA expression of PPARα and CPT1B as well as the gene or protein expression of CD36, SREBP1C, and FASN, which are involved in fat synthesis. PPARα can promote lipid metabolism, and activated PPARα can reverse lipid accumulation in cells [40]. Agonists of PPARα can regulate various stages of lipid and lipoprotein metabolism through transcription factors with the effect of lowering TG levels [41]. CPT1B is involved in fatty acid β-oxidation and is directly regulated by PPARα [42]. In the current investigation, PPARα and CPT1B were significantly decreased in both cell lines after BPS exposure, indicating that intracellular lipid metabolism was weakened. It is possible that BPS indirectly regulates the expression of CPTIB by inhibiting the expression of PPARα, leading to a decrease in cellular metabolism and the occurrence of lipid aggregation. SREBP1C is a lipogenic transcription factor that regulates intracellular lipid accumulation and plays an important role in the regulation of lipid metabolism [43]. FASN overexpression promotes hepatic TG accumulation as a key regulator of lipid metabolism [44]. CD36 acts as a lipid sensor, promoting fatty acid uptake [45,46] and possibly inducing dysregulation of lipid protein levels [47]. The mRNA expression of PPARα in SK-Hep-1 cells decreased, the expression of CD36 increased significantly after 24 h of BPS exposure, and the expression of SREBP1C and FASN was significantly reduced. Following 48 h of exposure, there was a marked increase in the expression of SREBP1C and FASN. Because the lipid synthesis genes SREBP1C and FASN were altered in SK-Hep-1 cells after exposure to BPS, this resulted in altered levels of the effector indicators TG and T-CHO as well. The trends in the levels of TG and T-CHO were consistent with the trends in the mRNA expression of SREBP1C and FASN, suggesting that the production of TG and T-CHO may be induced by SREBP1C and FASN in SK-Hep-1 cells. After 24 h of treatment, HepG2 cells’ expression levels of PPARα and CPT1B were markedly decreased. Although the expression level of CD36 was not significantly altered, the expression levels of SREBP1C and FASN were significantly increased, and this was consistent with the results of Western blotting. However, no significant changes in SREBP1C or FASN levels were observed after 48 h of BPS exposure. The results of qPCR and Western blotting indicated that the lipid metabolism of cells was inhibited and that lipid synthesis was promoted after BPS exposure. PPARα agonists are used in the clinic to reverse cholesterol transport and improve atherosclerotic lipoproteins [48], whereas activation of PPARα also ameliorates oxidative stress-induced organ damage [49]. The transcription factor, Krüppel-like factor 16, in the liver was found to target binding to PPARα to reduce oxidative stress in mice [50], and knockdown of PPARα causes lipid accumulation, whereas restoration of PPARα expression reduces mitochondrial oxidative stress [51]. In this study, inhibition of cellular PPARα expression by BPS resulted in enhanced oxidative stress, increased cholesterol transport, and increased levels of LDL-C, TG, and T-CHO. In summary, we hypothesize that BPS induces lipid metabolism disorders in HepG2 and SK-Hep-1 cells through oxidative stress by the following pathway: increased ROS and MDA content induces oxidative stress in cells, which directly or indirectly inhibits the expression of PPARα to regulate the expression of CPT1B, CD36, SREBPC, and FASN, and thus the cells undergo a disturbance in cholesterol metabolism, ultimately leading to cellular lipid accumulation. In this experiment, the mRNA or protein levels of SREBP1C and FASN related to lipid synthesis in HepG2 cells after 48 h of BPS exposure showed no discernible difference from those in the control group, and this may have been caused by the doubling of growth of HepG2 cells during culture in the presence of BPS without the exchange solution. It is also possible that the main pathway by which BPS causes lipid synthesis is the inhibition of PPARα expression, which reduces lipid metabolism and thus causes cellular lipid accumulation. In contrast, BPA, also an endocrine disruptor, increases SREBP1 mRNA expression to activate PPARγ which influences childhood obesity [52], and Di-2-ethylhexyl phthalate affects the normal metabolism of mouse liver through activation of LXR/SREBP1C and the PPARα signaling pathway [53]. This also illustrates the different mechanisms by which different chemicals affect different individuals.

In this study, although HepG2 and SK-Hep-1 cells are both human liver cancer cell lines, the biochemical indices and protein expression levels were not completely the same after BPS exposure, for example, after 48 h of BPS exposure, LDL-C levels were lower in SK-Hep-1 cells than in controls, and the difference between HepG2 cells and controls was not statistically significant, which further suggests that different individuals have different sensitivities to chemicals, which may undergo significant shifts over time, and may explain the differences between the two types of cells. This may result in different effects on different individuals in the population. Currently, the rate of obesity in the world is increasing, and BPS is widely used in various environmental media. Further studies are required to clarify the molecular mechanisms underlying the relationship among BPS, lipid accumulation, and obesity.

## Figures and Tables

**Table 1:** Real-time quantitative PCR primer sequences.

Gene	Primer	Sequences (5′ → 3′)
FASN	Forward	CGGAGTCGCTTGAGTATA
	Reverse	CACAGGGACCGAGTAATG
PPARα	Forward	CAAGTGCCTGTCTGTCGG
	Reverse	CAGGTAGGCTTCGTGGAT
CD36	Forward	ATTCTCATGCCAGTCGGA
	Reverse	TTTGCTGCTGTTCTTTGC
SREBP1C	Forward	ACAGTGACTTCCCTGGCCTAT
	Reverse	GCATGGACGGGTACATCTTCAA
CPT1B	Forward	AGACTGTGCGTTCCTGTA
	Reverse	TTGGAGACGATGTAAAG
GAPDH	Forward	CAGGAGGCATTGCTGATGAT
	Reverse	GAAGGCTGGGGCTCATTT

## Data Availability

The data that support the findings of this study are available from the corresponding author upon reasonable request.
